# The Influence of Beverages on Resin Composites: An In Vitro Study

**DOI:** 10.3390/biomedicines11092571

**Published:** 2023-09-19

**Authors:** Irina Gradinaru, Ana Lavinia Vasiliu, Alexandra Bargan, Laura Elisabeta Checherita, Bianca-Iulia Ciubotaru, Adina Oana Armencia, Bogdan Istrate, Cristina Gena Dascalu, Magda Ecaterina Antohe

**Affiliations:** 1 3rd Dental Medicine Department, Faculty of Dental Medicine, “Grigore T. Popa” University of Medicine and Pharmacy, 700115 Iasi, Romania; irina.gradinaru@umfiasi.ro (I.G.); magda.antohe@umfiasi.ro (M.E.A.); 2 Department of Functional Polymers, “Petru Poni” Institute of Macromolecular Chemistry, Aleea Grigore Ghica-Vodă, 41A, 700487 Iasi, Romania; vasiliu.lavinia@icmpp.ro (A.L.V.); anistor@icmpp.ro (A.B.); 3 2nd Dental Medicine Department, Faculty of Dental Medicine, “Grigore T. Popa” University of Medicine and Pharmacy, 700115 Iasi, Romania; checherita.laura@gmail.com; 4 Department of Inorganic Polymers, “Petru Poni” Institute of Macromolecular Chemistry, Aleea Grigore Ghica-Vodă, 41A, 700487 Iasi, Romania; ciubotaru.bianca@icmpp.ro; 5 1st Dental Medicine Department, Faculty of Dental Medicine, “Grigore T. Popa” University of Medicine and Pharmacy, 16 Universității Street, 700115 Iasi, Romania; 6 Department of Mechanical, Mechatronic and Robotic Engineering, The Faculty of Mechanics, “Gheorghe Asachi” Technical University, Bulevardul Profesor Dimitrie Mangeron 67, 700050 Iasi, Romania; bogdan.istrate@tuiasi.ro; 7 Department of Medical Informatics, “Grigore T. Popa” University of Medicine and Pharmacy, 16 Universității Street, 700115 Iasi, Romania

**Keywords:** dental composites, types of beverages, roughness, dental reconstructive therapy

## Abstract

Dental composites, through their structural diversity, represent the biomaterials frequently used in dental reconstructive therapy. The aim of our study was to observe the influence of different beverage environment conditions on seven types of obturation dental materials with different compositions. Our research focused on the surface modification analysis of the materials after the immersion in the different beverages; in this regard, we used the EDAX technique correlated with the energy-dispersive X-ray fluorescence (XRF). The pH of the drinks and that of the simulated saliva solution were determined by the titrimetric method, a sodium hydroxide solution 0.1 mol/dm^3^ was prepared and used for the titration. An amount of 5 mL of each analyzed solution was added to 15 mL of distilled water to obtain a dilution, to which 3 drops of phenolphthalein (as a color indicator—Phenolphthalein, 3,3-Bis(4-hydroxyphenyl)-1(3H)-isobenzofuranone, C_20_H_14_O_4_ Mw: 318.32, purchased from Merck) were added for each analysis. For each solution, the experiment was repeated three times in order to obtain accurate results. The results of our study materialized into a real plea for modifying the patients’ behavior in terms of diet and preferences for acidic drinks, so that their quality-of-life valence can be improved by keeping the composite materials in a long-term unalterable state on the one hand; on the other hand, systemic damage can be prevented as well.

## 1. Introduction

Dental composites are biomaterials frequently used in various types of restorations due to their biomechanical properties correlated with optimal aesthetics and not least their ability to establish durable bonds with dental hard tissues. The clinical performance of dental restorations made of composite materials depends on a number of factors, including the structure of the restorative biomaterial and the manner in which it can be degraded by altering its mechanical properties such as wear resistance, adhesion, integrity of the tooth-restoration interface, and changes in roughness [1,2].

The alteration of the surface condition of composite materials is a risk factor for oral health, and these changes are in accordance with the patient’s lifestyle, especially with regard to eating habits [3].

Extrinsic factors that may affect the surface condition of composite materials include, in most cases, the consumption of acidic foods and energy drinks [4,5].

Intrinsic factors that may affect the surface condition of composite materials include chronic gastrointestinal pathology, anorexia, bulimia, and clinical situations leading to a reduction in pH in the oral environment [6]. The consequences of the action of intrinsic or extrinsic factors on composite materials intended for dental restorations are reflected in increased surface roughness of the composite, making it susceptible to wear.

The degradation of composite materials remains a key Issue in dental practice, even in the context of spectacular technological developments and significant improvements in the physical and mechanical properties of dental composites. Different types of beverages can promote surface erosion of dental composites; thus, the increased consumption of carbonated energy drinks raises questions about the erosive potential of these beverages on dental tissues on the one hand and on the maintenance of the integrity of restorative materials over time on the other [7].

On the one hand, contemporary society confronts us with a line of healthiness that considerably increases the quality of life of patients, while, on the other hand, it offers an extremely varied range of drinks and foods that can have negative effects on the general balance of the body, and can also act in oral territory [8].

Literature data provide us with sufficient information on the influence of chemical and thermal factors on the long-term clinical success of dental composite restorations.

Chemical factors play a particularly important role in the surface degradation process of composite resins, which is reflected in the change in surface roughness of these biomaterials [9].

Choosing the composite material of choice for dental restorations in the context of the particularities of each clinical case has always been a real challenge for the practitioner, which is why the literature provides us with numerous studies analyzing the structural changes of composite materials under the influence of different factors. Thus, most studies are interdisciplinary, in which scanning electron microscopy (SEM) provides the most details about the adhesive interface, and energy dispersive X-ray analysis (EDX) allows a semi-quantitative evaluation of the chemical elements on the surface of the substrate analyzed. EDX has been widely used in engineering and chemistry but less so in dentistry, with interdisciplinary collaborations exploiting these methods to obtain results with profound clinical impact, the most important implications of the end result of these studies being related to improving the quality of life of patients [10].

In their studies, Show and Smith quantified the erosive potential of beverages, obtaining high values for juices, with orange juice, grapefruit juice, and apple juice in first place, and medium values for Coca Cola, sparkling orange juice, and white wine [11].

The prevalence of mechanical or aesthetic properties of these biomaterials is fully in accordance with the location of the tooth to be restored, in conjunction with the type of static and dynamic occlusion of the patient. The long-term maintenance of the integrity of dental restorations with composite materials depends on a cumulative factor, in which the preferred food or drink may influence the structure of these composite materials found in the different types of dental fillings. Dental fillings are constantly exposed to different factors acting in the oral cavity that can intervene to alter the basic properties of restorative materials, depending on the time of action as well as the frequency of their intervention. Of the wide range of factors that can alter the structure of composite materials, chemical factors can lead to erosive defects in both the dual tissues of teeth and composite materials [12].

It is known that some of the most popular drinks have a different erosive potential depending primarily on the pH as well as the titration acidity [13].

A current societal trend is reflected in international studies that indicate an increase in the consumption of potentially erosive beverages among children, with data from the American Academy of Pediatric Dentistry report indicating that the consumption of sweetened beverages in the United States has increased by 300% over the past 20 years. It is known that the adverse effects of potentially erosive sweetened beverages are reflected in two registers, at the level of the general status through the appearance of obesity and digestive pathology, and at the level of the oral cavity through the lowering of pH, favoring the development of various types of erosive defects in the enamel, which creates the premises for the overgrowth of microorganisms that metabolize sugar, with the immediate reactions being demineralization of the enamel and the appearance of carious pathology [14].

We considered the aspects that we proposed to develop in this study to be important both in terms of the therapeutic decision to choose the restorative composite material and in terms of the patients’ lifestyle, so that from the results obtained, clear trajectories can be outlined both in terms of the choice of restorative materials in the context of the analysis of the patient’s lifestyle, without circumventing the usual criteria of choice and in terms of education in order to achieve an optimal health for patients, thus significantly increasing their quality of life.

The need for this study was derived from an eloquent clinical reality, namely the damage of composite fillings with implications on the functionality of the dental system, the damage being different depending on the location of the filling; a particularly important aspect on which we based our study was the fact that many of the beverages that our patients consume have a negative impact on oral health. So, this study is a plea for sanogenity through the results obtained using the methods represented by EDAX and XRF.

Our study was focused on the analysis of structural changes, prevalent in the surface state of different types of composite materials by means of the EDX technique correlated with XFD, under the influence of some drinks that most patients consume without assessing the risk they may have on dental restorative materials. For this, the seven materials were purchased and prepared as per the producer’s instructions in a clinical environment, maintaining the proper conditions accordingly.

The null hypothesis of the study was the idea that the surface condition of the dental composites involved in the different types of restorative fillings may not be affected by the beverages consumed.

## 2. Materials and Methods

### 2.1. Experimental Part

Seven commercially available dental obturation materials were purchased and prepared as per the producer’s instructions in a clinical environment, maintaining the proper conditions accordingly. The material samples were immersed in the different beverages and in the simulated saliva solution and kept for 24 h in contact. After this, the materials were taken out and analyzed.

The pH of the drinks and that of the simulated saliva solution were determined by the titrimetric method, a sodium hydroxide solution 0.1 mol/dm^3^ was prepared and used for the titration. Then, 5 mL of each analyzed solution was added to 15 mL of distilled water to obtain a dilution to which 3 drops of phenolphthalein (as a color indicator—Phenolphthalein, 3,3-Bis(4-hydroxyphenyl)-1(3H)-isobenzofuranone, C_20_H_14_O_4_ Mw: 318.32, purchased from Merck) were added for each analysis. For each solution, the experiment was repeated three times in order to obtain accurate results.

The results were calculated using the formula:

$$X_{TV}=\frac{V\times c\times M}{m}\times \frac{V_{0}}{V_{1}}\times 0.1$$

where:

V—volume of sodium hydroxide solution 0.1 mol/dm^3^ used for titration, measured in cm^3^;
V_0_—the total volume of the solution to be analyzed obtained from the amount of product taken for analysis, measured in cm^3^;
V_1_—the volume of the solution to be analyzed, taken for determination, in cm^3^;
c—the molar concentration of the sodium hydroxide solution, mol/dm^3^;
m—mass of the product taken for analysis, g;
M—molar mass of the organic acid that predominates in the analyzed product, g/mol, in this case citric acid, contained by every solution, M (1/3 C_6_H_6_O_7_) = 64 (Table 1).

The characteristics of the environment and of the beverages analyzed from a structural point of view are shown in Table 2.

The parameters characterizing the composite materials analyzed from a structural point of view are given in Table 3.

### 2.2. Methods

Morphological characterization and content analysis through EDAX: Surface morphology and elemental composition of the analyzed samples were evaluated with the help of a Verios G4 UC Scanning electron microscope (Thermo Scientific, Pardubice, Czech Republic) equipped with Energy Dispersive X-ray spectroscopy analyzer (Octane Elect Super SDD detector, Pleasanton, CA, USA). The samples were coated with 10 nm platinum using a Leica EM ACE200 Sputter coater to provide electrical conductivity and to prevent charge buildup during exposure to the electron beam. SEM investigations were performed in High Vacuum mode using a secondary electron detector (Everhart-Thornley detector, Kansas City, MO, ETD) at accelerating voltage of 5 kV.

The energy-dispersive X-ray fluorescence (XRF), an EX-2600 X-Calibur SDD system, was used in order to determine the presence and ratio of the elements (Si, Ba, Ca, P, etc.) in the composition of samples, and revealed in spectral form. Our samples were first analyzed as such, without being immersed in the simulated salivary fluid media or beverages, and then after 24 h immersion.

## 3. Results

### 3.1. Scanning Electron Microscopy

**Figure 1:**
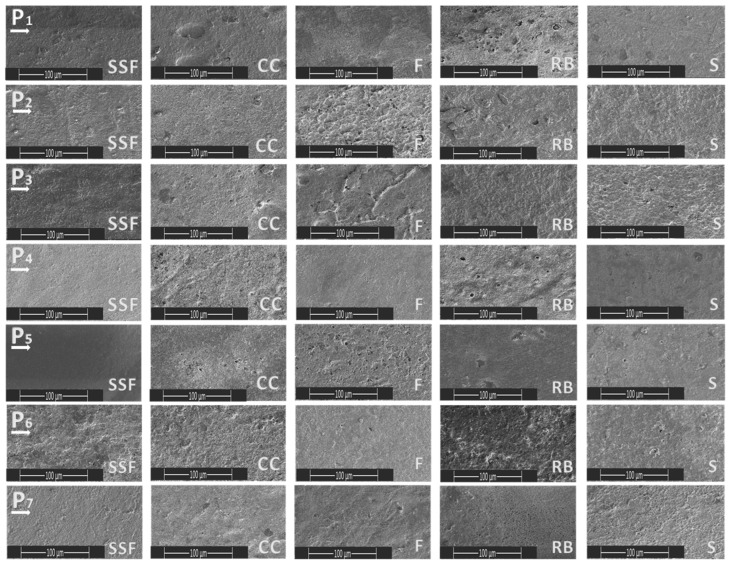
SEM images taken after 24 h of immersion in the different media (100 µm).

**Figure 2:**
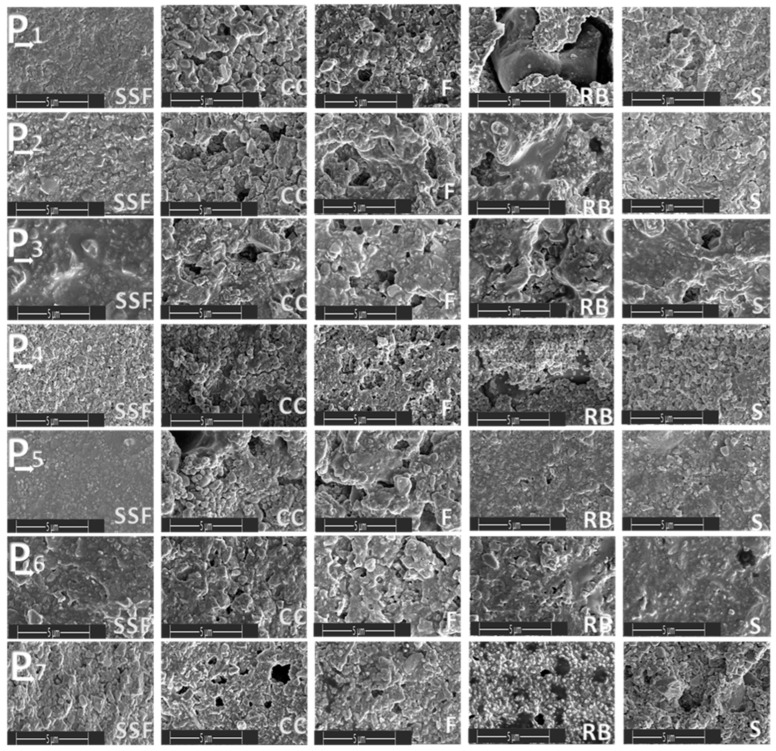
SEM images taken after 24 h of immersion in the different media (5 µm).

Seven material sample/dental fillings were immersed in five different media (SSF—simulated salivary fluid, F—Fanta, CC—Coca Cola, S—Schweppes, and RB—Red Bull) for 24 h and were analyzed morphologically. The materials immersed in artificial saliva were used as reference samples. In all four cases in which sour drinks were used, a change in the roughness of the material was observed, with minor changes in the case of immersion in Schweppes. Figure 1 and Figure 2 show the morphology of the surfaces of the samples at 100 µm and 5 µm, respectively.

SEM images at a scale of 100 microns highlighted the comparative appearance of surface morphology in the five immersion environments for the experimental samples. For the 24 h immersion interval, the surface appearance was generally uniform, very little affected, with the local appearance of small uneven pores distributed in the surface of the samples (P1-RB, P2-F, P3-F, P4-CC, P4-RB, P5-CC, P5-F).

SEM images at a scale of five microns highlighted the comparative appearance of surface morphology after immersion in specific media for 24 h. The surface appearance showed many pores of varying sizes as well as the presence of microcracks.

Taking the samples in point form, we can specify the following:

- Sample P1: the accentuated degradation was identified in the case of the RB medium, where the presence of an inclusion of foreign nature in the structure of the material was also observed.
- Sample P2: the accentuated degradation was identified in the case of CC and F medium, where the presence of unevenly distributed micropores was also observed.
- Sample P3: marked degradation was identified for the RB medium and moderate degradation for the other immersion media, but also the presence of unevenly distributed micropores and variable sizes.
- Sample P4: marked degradation was identified for the RB medium, and moderate degradation for the F immersion medium, where the presence of unevenly distributed micropores was also observed.
- Sample P5: pronounced degradation was identified in the case of medium CC and F, where the presence of unevenly distributed micropores was also observed.
- Sample P6: pronounced degradation was identified in the case of medium F, where the presence of pores was to a small extent.
- Sample P7: medium degradation was identified in all immersion media, where the presence of unevenly distributed micropores was also observed.

### 3.2. Energy Dispersive Analysis X-ray

**Figure 3:**
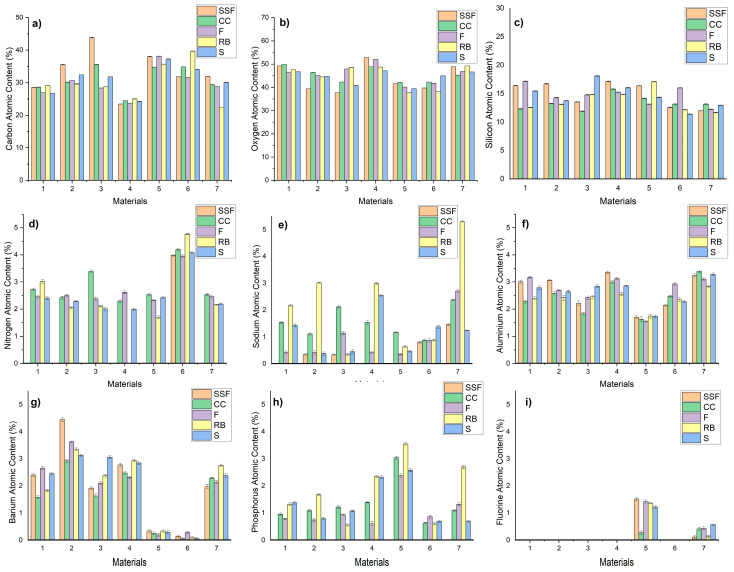
The atomic content of the materials(**a**–**i**). (**a**) Carbon atomic content (%); (**b**) Oxygen atomic content, (**c**) Silicon atomic content (%); (**d**) Nitrogen atomic content (%); (**e**) Sodium atomic content (%); (**f**) Ammonium atomic content (%); (**g**) Barium atomic content (%); (**h**) Phosphorus atomic content (%); (**i**) Fluorine atomic content (%).

The atomic content of the materials was analyzed after 24 h of immersion in the different media and is represented in Figure 3.

**Figure 4:**
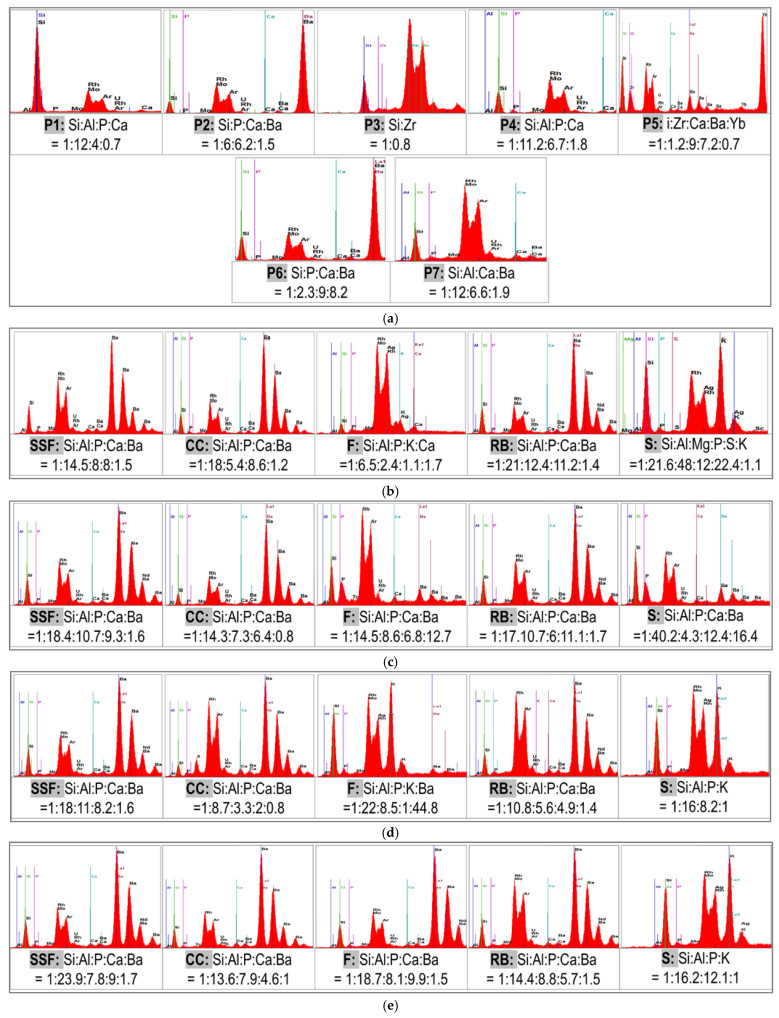

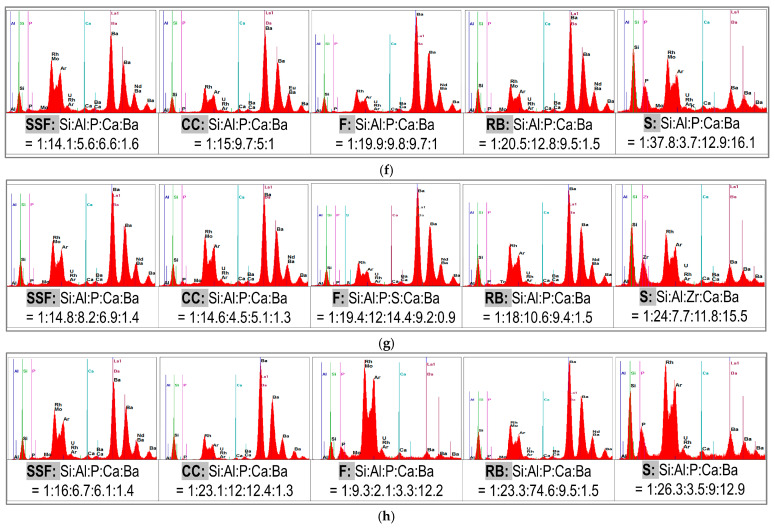
XRF analysis: (**a**) Initial spectra registered before the immersion in different beverages; (**b**–**h**) XRF analysis for P1–P7.

The EDS analyses were carried out in three different areas, and so, Figure 4 shows the mean percentage value of the chemical components and the standard deviation. The mean standard deviation for each element is as follows:

C: ±0.052; O: ±0.044; N: ±0.034; Na: ±0.039; Al: ±0.05;

Si: ±0.056; Ba: ±0.049; P: ±0.039; F: ±0.015.

In terms of carbon content, in samples 1, 4, and 5, it remained similar for the artificial saliva and beverages tested. There was a decrease in carbon content in sample 3 dipped in all four drinks tested, which was more pronounced for F and RB. A decrease was also observed for samples 2 and 7, but a smaller decrease. In the case of sample 7, the carbon content decreased more drastically when immersed in RB. In the case of sample 6, the C content decreased when immersed in F.

Oxygen content was similar to immersion in artificial saliva and carbonated beverages for samples 1, 5, and 7. On the other hand, there was an increase in oxygen content when immersed in carbonated beverages for samples 2, 3, and 6, which was more pronounced for samples 2 and 3. In sample 2, the highest oxygen content was observed in CC followed closely by F, RB, and S, and in sample 3, the highest oxygen content was observed in F and RB.

Silicon content was similar to saliva and carbonated beverage immersion in sample 7. A slight increase was observed for sample 3 dipped in S and sample 6 dipped in F, respectively, a decrease for sample 1 dipped in CC or RB, sample 5 dipped in F, and sample 2 dipped in all four beverages tested.

Nitrogen was identified only in the case of sample 6 dipped in artificial saliva, at a level that remained similar to that of immersion in the tested carbonated drinks. For the other biomaterials, nitrogen was not identified at all when immersed in artificial saliva, but instead was identified when immersed in carbonated beverages—hence, this chemical element was taken up exclusively from the beverages. The highest amount of nitrogen was observed in sample 3 immersed in CC—the other results were comparable; no nitrogen was identified in sample 4 immersed in RB.

Sodium was not identified at all when immersed in artificial saliva in samples 1, 4, and 5, being identified in very low percentages in samples 2 and 3 and slightly higher in samples 6 and 7; in contrast, when immersed in carbonated beverages, the percentage of sodium increased and was present in all seven samples tested. The highest values were observed for sample 7 immersed in RB, respectively, CC or F, sample 4 immersed in RB and S, samples 1 and 2 immersed in RB, with observable sodium percentages also in samples 1, 3, and 4 immersed in CC. However, it can be stated that the highest sodium intake was obtained when samples are immersed in RB.

Aluminum content is similar in artificial saliva and carbonated beverages in samples 1, 5, and 7, increased slightly in S-immersed sample 3 and F-immersed sample 6, and decreased slightly in CC or RB-immersed sample 1 and RB-immersed sample 4.

The highest Barium content was found in sample 2 immersed in artificial saliva—this content decreased when sample 2 was immersed in carbonated drinks. In the case of samples 1, 4, and 7, Barium was identified when immersed in artificial saliva, but also in carbonated beverages—in similar percentages; for sample 3, Barium content increased when immersed in S compared to the artificial saliva situation. For samples 5 and 6, Barium was detected only in negligible amounts.

Phosphorus was taken up entirely from carbonated beverages—not identified for any of the samples tested when immersed in artificial saliva. Instead, phosphorus was detected in carbonated beverage immersion, in the highest percentages in the case of sample 5. The highest percentages were observed in RB immersion of sample 5, but also in the other samples (4, 7, and 2); CC immersion also led to increased phosphorus percentages, especially for sample 5, but also for the others, as did S immersion.

Fluorine was not identified at all in samples 1, 2, 3, 4, and 6, and in negligible amounts in sample 7. In sample 5, it was identified in artificial saliva immersion and remained at similar levels in F, RB, or S immersion, but decreased drastically in C immersion—which, therefore, removed it from the biomaterial structure.

### 3.3. Energy-Dispersive X-ray Fluorescence (XRF)

The results and calculated ratios of the elements present in the different materials are shown in Figure 4.

The XRF analysis can reveal more quantitative chemical detection of the elements contained by the materials, having a better detection limit than the EDAX analysis and being an in-depth analysis.

The XRF analysis reinforced the results indicated by the EDS analysis, by confirming in each sample the chemical elements of the Si element. The average XRF results in different environments contained standard deviation values, and the identified chemical elements were related to the element Silicon.

In general, the same composition elements were identified and are comparable with the EDS analysis.

## 4. Discussion

Surface condition analysis provides relevant data on the role of erosive factors in an individual diet, i.e., acidic drinks, on the properties of composite materials, aspects that accentuate the surface degradation state of composites are influenced by the frequency and quantity of these products that can influence the deterioration of the mechanical properties of composite materials.

Relevant studies [13,14,15,16,17] on a number of eight samples made of composite materials anchored in the micro hybrid and nanohybrid register provide useful information about the influence of four beverages, sparkling water, Red Bull, Coca Cola, and orange juice, on the structure of the analyzed composites. The Vickers microhardness test and flexural strength test were used to evaluate the structural change of the composites analyzed. The results of the study support the decrease in microhardness of dental composite resins, with statistically significant results in the quantification of the flexural strength of the Gradia composite directly above. In our studies, the Gradia composite showed a marked degradation in case of immersion in Fanta, aspects that can be supported by the Bio behavior of the material according to its structure. In our study, we noticed an average degradation of the surface condition for Carisma Classic in all the beverages analyzed behavior that can be explained by the new Microglass^®^ II filler technology.

It was clearly observed that the immersion medium interacted first with the binder and then with the composite, thus producing an accelerated degradation, which negates the null hypothesis.

The tendency is for degradation at the interface of the composite component grains. Thus, in the case of sample P1, the more pronounced degradation is for immersion in CC and RB, sample P2—F and RB, sample P3—F, sample P4—CC and RB, sample P5—F and RB, sample P6-RB, and sample P7—S.

Predominantly, oxygen, carbon, and silicon are elements present in all seven pre-immersed samples, as well as aluminum in a smaller percentage. Nitrogen was present for sample P6 immersed in all mediums, but not for the other six (P1, P2, P3, P4, P5, and P7) from the phosphate-buffered solution. Sample P4 immersed in RB also did not have any nitrogen atomic content. For sodium, samples P2, P3, P6, and P7 presented traces in all mediums, but samples P1, P4, and P5 had it in all other mediums except for phosphate buffer. Barium was also present, except for samples of P6 in CC and S. Phosphorous was present in all analyzed samples from all mediums, except from buffer solution. Fluoride was only present in samples from P5 from all mediums and for some of P7 from CC, F, and S.

The data of our study in terms of surface state alteration are superimposable as a type of structural damage in the presence of acidic beverages on the studies conducted by Yanikoglu et al. [18], Tanthanuch [19], and Aliping-McKenzie et al. [20], highlighting the negative impact that acidic beverages can have on the structure of dental composites.

The results indicate the effect of the surface condition in conjunction with the evaluation of the atomic content of the materials under the action of carbonated beverages, all these results being significant in comparison with the structure of artificial saliva, the effect of the surface condition is in full agreement with the properties of the materials, noting their final behavior, which is supported by the contribution of different types of fillers and the prevalence of these fillers. Our research has shown that the structure of biomaterials is mandatory for their behavior in the presence of acidic beverages; thus, micro-hybrid materials show significant changes in surface condition. It is important to note that the differences between the tested materials differ according to the size of the filler in conjunction with the chemical composition.

Our study argues for the impact that Red Bull components have on the disruption of the surface state of composite materials and the disruption of atomic equilibrium, results superimposable on the studies, by Erdemir et al. [21,22,23,24,25]. The negative effects of Red Bull on the structure of composite materials agree with their structure, it is followed by Coca Cola and Fanta.

Good results in terms of surface state behavior in the presence of acidic beverages were given by Admira Fusion composites, behavior supported by the presence of an ORMOCER-based restorative system involving very low shrinkage stress and reality, which is a micro-hybrid material that has a BIS-GMA-based structure having a total filler loading of 81%.

It is extremely important that the therapeutic decision in the choice of restorative materials is dictated by the patient’s lifestyle, the location of the tooth to be restored, the type of occlusion, and, last but not least, the bio-material structure chosen, which must be appropriate to the biomechanical and aesthetic requirements [26,27,28,29,30,31,32].

## 5. Conclusions

Interdisciplinary studies provide evaluation support in clinical territory; thus, EDAX and XRF methods have proven to be extremely useful in evaluating surface state and atomic composition changes in composite biomaterials, whose behavior is individualized in the presence of different types of carbonated beverages.

The study carried out leads to the clear conclusion that all the beverages tested modified the surface condition of the samples made of composite materials.

The tendency is for degradation at the interface of the composite material component grains. Thus, in the case of Brilliant NG, the more pronounced degradation was for the immersion in Coca Cola and Red Bull, for Es Com 100—Fanta and Red Bull, for Admira Fusion—Fanta,—Coca Cola and Red Bull, for reality—Fanta and Red-Bull, for GC GRADIA Direct—Red Bull, and for Charisma Classic—S Schweppes.

Silicon content is similar to saliva and carbonated beverage immersion in sample 7, and Charisma Classic, respectively.

Fluorine was identified in negligible amounts in the case of sample 7 and Charisma Classic, respectively, without being found in the other samples. In Optishade, it was identified in artificial saliva immersion and is maintained at similar levels in Fanta, Red Bull, or Schweppes immersion, decreasing drastically in Coca Cola immersion—which, therefore, removes it from the biomaterial structure.

The results of our study materialize into a real plea for modifying patients’ behavior in terms of diet and preferences for acidic beverages, so that their quality-of-life valence can be improved by maintaining composite materials in a long-term unalterable state on the one hand; on the other hand, systemic damage can be prevented as well.

## Figures and Tables

**Table 1:** Titrimetric acidity values (X_TV_) obtained for the studied beverages.

Media/Beverage	X_TV_ (%)
SSF	0.221 ± 0.0010
CC	0.370 ± 0.0012
F	0.650 ± 0.0011
RB	1.123 ± 0.0015
S	0.761 ± 0.0010

**Table 2:** Materials.

Sample	Beverage/Medium	Content (from Package)	Measured pH Value	Source
SSF	Simulated salivary fluid	Disodium hydrogen phosphate 2.382 g Potassium dihydrogen phosphate 0.19 g Sodium chloride 8.00 g Distilled water Up to 1 litre	6.8	preparation
CC	Coca Cola zero sugar	Water, carbon dioxide, colour E 150d, sweeteners, cyclamates, acesulfame-K and aspartame, acidifier phosphoric acid, natural flavours, caffeine, acidity regulator sodium citrate, phenylalanine, salt 0.02 g in 100 mL	2.4	Bucharest, Romania
F	Fanta	Water, orange juice from concentrate 3%, carbon dioxide, acidifiers, citric acid and malic acid, sweeteners: cyclamates, acesulfame-K, glycosides derived from steviol and E 959, natural orange flavours with other natural flavours, preservative potassium sorbate, antioxidant: ascorbic acid, stabiliser guar gum, colouring carotenes. 0.3 g sugar/100 mL	3	Bucharest, Romania
RB	Red Bull	Carbonated water, sucrose, glucose, acidifier (citric acid), taurine (0.4%), acidity regulators (sodium carbonates, magnesium carbonates), caffeine (0.03%), vitamins (niacin 8 mg/100 mL, pantothenic acid 2 mg/100 mL, vitamin B6 2 mg/100 mL, vitamin B12 2 μg/100 mL), flavourings, colourings (plain caramel, riboflavin). 0.1 g salt and 11 g/100 mL sugars.	3	Fuschl am See, Austria
S	Schweppes	Water, sugar, carbon dioxide, lemon juice (1.5%), lemon extract, acidifier citric acid, flavourings including quinine, preservative potassium sorbate, stabilisers E 1450 and E 445. 12.6 g/100 mL sugars	2.5	Bucharest, Romania

**Table 3:** Structure of the composite materials analyzed.

No. of Sample	Name	Company	Material Type	Filler Size	Characteristics	Filler Type
P1	Brilliant NG	Coltene, Switzerland	Nano-Hybrid	range of particle size: 0.01–2.5 μm	radiopaque, filler content by volume: 65% filler content by weight: 80%	Dental glass, amorphous silica
P2	Es Com 100	Spident Co., Ltd., Republic of Korea	Nano-Hybrid	microparticles ranging in size from 10 nm to 10 microns	low polymerization shrinkage, and superior compression strength	Bis-GMA, UDMA Barium glass, Silicone dioxide
P3	Admira fusion	Voco, Germany	Nano-Hybrid Ormocer	filler particles have 1μm of size	restorative system based on ORMOCER^®^ has the lowest polymerization shrinkage (1.25% by volume) and, coupled with this, very low shrinkage stress	- inorganic silicon dioxide and polymerized organic units, like zirconium dioxide (ZrO_2_) and silicon dioxide (SiO_2_)
P4	Reality	Schulzer, Germany	Micro-hybrid	size of inorganic particles: 0.05–1.5 microns	The total fill load is 81% and the total fill volume is 65%. Is distinguished by high stability, abrasion resistance, excellent polishing properties	BIS-GMA-based
P5	Optishade	Kavo-Kerr, Germany	Nano-hybrid based on Adaptive Response technology (ART)	Filler particle size less than 50 nm	Better resistance to chipping and fracturing Optimum radio opacity and translucency	Selection of chemically infused mixed oxides, pre-polymerized filler, barium, glass filler, silica and ytterbium trifluoride
P6	GC GRADIA Direct	GC E, Belgium	Micro-hybrid	Silica—0.85 µm	Has a high resistance to fracture and a high degree of elasticity	Barium silicate glass, silica dioxide, pre-polymerized filler
P7	Charisma Classic	Heraeus-Kulzer (Wehrhein, Germany)	Micro-hybrid composite	particle size of 0.005–10 μm	Excellent finishing and polishing properties facilitate a high surface lustre the new filler technology offers an intrinsic shade brilliance and increased colour adaption of up to 56%	BIS-GMA matrix Barium Aluminium Fluoride glass Pre-polymerized filler

## Data Availability

The data presented in this study are available on request from the corresponding author.
